# Supplementary material for: Identification of photoperiod-induced specific miRNAs in the adrenal glands of Sunite sheep (Ovis aries)
Source: Front Vet Sci. 2022 Jul 22;9:888207. doi: 10.3389/fvets.2022.888207 (PMC9354845; doi:10.3389/fvets.2022.888207)
Supplement: Supplementary file 1 [file Data_Sheet_1.ZIP › Supplementary file/miRDeep2 file.zip!/miRDeep2 file/Know_Structure/SPLP42A2/oar-mir-539.pdf]

miRBase precursor : oar-mir-539
Total read count : 1306
oar-miR-539-5p read count : 4
oar-miR-539-3p read count : 1302
remaining reads : 0

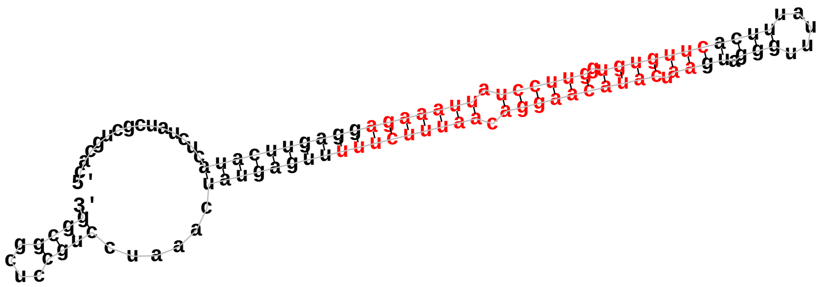

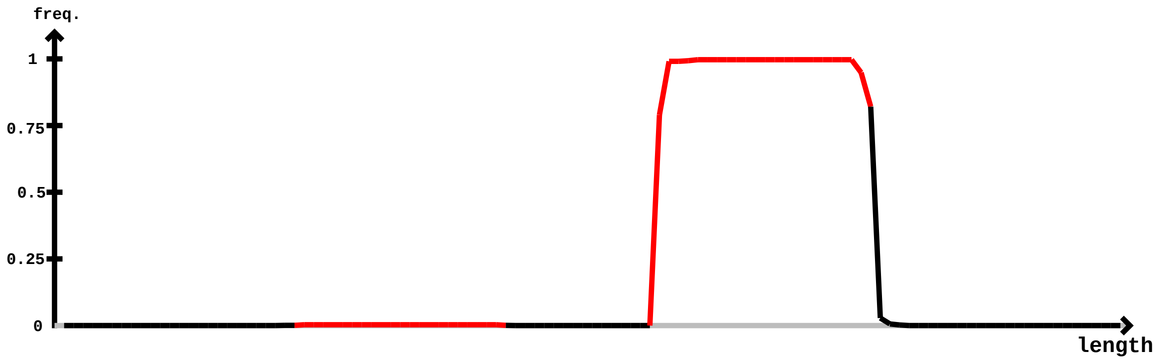


```
                                                                oar-miR-539-3p
                         oar-miR-539-5p
5'- cacgucgcuaucucauacuugaggagaaauuauccuugguguguucacuuuauuugggaugaaucauacaaggacaauuucuuuuugaguaucaaauccugccucggcgg  -3'   exp
    ..............(((((((((((((((((.((((((.(((((((((((.....))).)))).)))))))))).)))))))))))))))))......((((....))))   reads   mm   sample
    ......................ggagaaauuauccuugguguguu.................................................................   1       0    seq
    ........................agaaauuauccuugguguguu.................................................................   2       0    seq
    ........................agaaauuauccuugguguguuc................................................................   1       0    seq
    .............................................................aaucauacaaggacaauuucu............................   62      0    seq
    .............................................................aaucauacaaggacaauuucuu...........................   156     0    seq
    .............................................................aaucauacaaggacaauuucuuu..........................   801     0    seq
    .............................................................aaucauacaaggacaauuucuuuu.........................   13      0    seq
    ..............................................................aucauacaaggacaauuucu............................   1       0    seq
    ..............................................................aucauacaaggacaauuucuu...........................   11      0    seq
    ..............................................................aucauacaaggacaauuucuuu..........................   233     0    seq
    ..............................................................aucauacaaggacaauuucuuuu.........................   15      0    seq
    ..............................................................aucauacaaggacaauuucuuuuu........................   2       0    seq
    ................................................................cauacaaggacaauuucuuuu.........................   1       0    seq
    ................................................................cauacaaggacaauuucuuuuu........................   2       0    seq
    .................................................................auacaaggacaauuucuuu..........................   1       0    seq
    .................................................................auacaaggacaauuucuuuuu........................   1       0    seq
    .................................................................auacaaggacaauuucuuuuug.......................   3       0    seq
```
